# Inhibition of Mcl-1 expression by citrate enhances the effect of Bcl-x_L_ inhibitors on human ovarian carcinoma cells

**DOI:** 10.1186/1757-2215-6-72

**Published:** 2013-10-08

**Authors:** Hubert Lincet, Perrine Kafara, Florence Giffard, Edwige Abeilard-Lemoisson, Maryline Duval, Marie-Hélène Louis, Laurent Poulain, Philippe Icard

**Affiliations:** 1 Normandie Univ France, Caen, France; 2 BioTICLA EA 4656, Caen F-14032, France; 3 Centre de Lutte Contre le Cancer François Baclesse, Avenue du Général Harris, Caen, BP5026 14076, Cedex 05, France; 4 Service de Chirurgie Thoracique, Centre Hospitalier Universitaire de Caen Basse-Normandie, Avenue de la Côte de Nacre, Caen 14000, France

**Keywords:** Mcl-1, Citrate, Bcl-x_L_, Ovarian cancer, Cell death

## Abstract

The inhibition of two major anti-apoptotic proteins, Bcl-x_L_ and Mcl-1, appears essential to destroy chemoresistant cancer cells. We have studied their concomitant inhibition, using ABT 737 or siRNA targeting XL1 and citrate, a molecule which reduces the expression level of Mcl-1.

Two cisplatin-chemoresistant ovarian cell lines (SKOV3 and IGROV1-R10) were exposed to ABT 737 or siRNA targeting XL1 and citrate at various individual concentrations, or combined. Cell proliferation, cell cycle repartition and nuclear staining with DAPI were recorded. Western blot analyses were performed to detect various proteins implied in apoptotic cell death pathways.

Mcl-1 expression was barely reduced when cells were exposed to citrate alone, whereas a mild reduction was observed after ABT 737 treatment. Concomitant inhibition of Bcl-x_L_ and Mcl-1 using ABT 737 or siXL1 associated with citrate was far more effective in inhibiting cell proliferation and inducing cell death than treatment alone.

Given that few, if any, specific inhibitors of Mcl-1 are currently available, anti-glycolytic agents such as citrate could be tested in association with synthetic inhibitors of Bcl-x_L_.

## Introduction

Chemoresistance increases the seriousness of cancer, since, in the absence of effective chemotherapy, other treatment modalities (surgery, radiotherapy) are often doomed to failure, especially when cancer is diagnosed at an advanced stage. For ovarian cancer, which is the fifth most frequent cause of tumour death in women, the 5-year survival rate for stage III and IV disease (representing around 75% of cases) is approximately 20% - 30%. These disappointing results are mainly due to intrinsic chemoresistance or to progressively acquired resistance, despite a good response to surgery and to first-line platinum-based chemotherapy [1,2].

When active, chemotherapies lead to the apoptotic death of cancer cells [3,4]. Apoptosis is controlled by members of the BCL-2 family, which promote or inhibit this process [5]. The main anti-apoptotic members are Bcl-2, Mcl-1 and BCL-x_L_, whereas the main pro-death members are Bax, Bak, Bad and Bim [6,7]. Overexpression of anti-apoptotic Bcl-2 family proteins has been reported as contributing towards cell survival and drug resistance in a variety of human malignancies [8,9]. In various cancers, Bcl-x_L_ and Mcl-1 are frequently overexpressed [8], suggesting that resistance to apoptosis could imply these two key anti-apoptotic factors. Furthermore, Bcl-x_L_ and Mcl-1 are frequently associated with chemotherapeutic resistance and relapse [9,10]. Therefore, anti-apoptotic strategies, such as concomitant inhibition of Bcl-x_L_ and Mcl-1 are expected to play a potentially important role in the near future to overcome drug resistance in certain cancers [9,11,12]. Given that Bcl-x_L_ inhibitors are currently under clinical evaluation (e.g. BH3 mimetic compounds such as antimycin A3, gossypol, HA14-1 [13] or ABT-737) [14-16]), and that there are few or no available specific inhibitors of Mcl-1 [17], the discovery of new Mcl-1 inhibitors is of crucial interest.

Citrate is an intermediate of the tricarboxylic acid cycle (TCA), which acts as an “energy gauge” inside the cell since, when in excess, it inhibits glycolysis by allosteric retro-control on phosphofructokinase (PFK). This enzyme catalyses the first irreversible reaction of glycolysis and constitutes the main control checkpoint of this pathway. Because glycolysis furnishes essential intermediates (such as ribose, glycerol, serine …) required for cancer cell proliferation and a significant share of ATP, especially in hypoxic conditions [18-21], its blockage could arrest cancer cell growth [22-25]. When citrate is abundant, PFK activity is virtually switched off, leading to a slowdown in TCA. Interestingly, citrate leads to early down-expression of Mcl-1 [13;28].

ABT-737 is a small chemical molecule that mimics the direct binding of Bad to Bcl-2, Bcl-x_L_ and Bcl-w, but not to Mcl-1 [26]. It has demonstrated preclinical activity as a single agent in a wide variety of cancer cells, such as hematopoietic cell lines [27-30] and, to a lesser extent, solid tumour cell lines [32;34–37]. However, ABT-737 is unable to induce cell death as a single agent in other tumour models, such as prostate cancer [38]. This relative resistance has previously been linked to a persistence of Mcl-1 expression [38–40]. Our work confirms the primordial importance of the concomitant inhibition of the two key anti-apoptotic proteins, Mcl-1 and Bcl-x_L_. Citrate, which reduces the expression of Mcl-1, dramatically increases cell death in chemoresistant human ovarian carcinoma cell lines exposed to interfering RNA targeting Bcl-x_L_ or ABT-737.

## Materials and methods

### Cell line and culture

The SKOV3 cell line was established from a human ovarian adenocarcinoma and was obtained from ECACC (Sigma-Aldrich, Saint Quentin Fallavier, France). The variant, the highly chemoresistant cell line IGROV1-R10, was established through a protocol that mimics the clinical protocol of CDDP treatment leading to a complete CDDP-refractory cell state [31]. These two cell lines were grown in an RPMI medium supplemented with 4,500 mg/L glucose, 2 mM Glutamax™, 1 mM sodium pyruvate, 10% foetal calf serum, 33 mM sodium bicarbonate (Invitrogen, Cergy-Pontoise, France). Cells were maintained at 37°C in a 5% CO_2_ humidified atmosphere and split twice a week by trypsinisation.

### Chemicals and schedule for treatment

#### siRNA synthesis and transfection

All siRNAs used in these studies were chemically synthesised by Eurogentec (Liege, Belgium) and received as annealed oligonucleotides. The sequence for the double-stranded RNA used to inhibit XL1 expression (noted siXL1) is: sense 5′-AUUGGUGAGUCGGAUCGCA-3′ and anti-sense 5′-UGCGAUCCGACUCACCAA-3′. siXL1 selectively targets Bcl-x_L_ mRNA, but not Bcl-x_S_ mRNA. The sequence for the control siRNA siCTRL is: sense 5′-GACGUAAACGGCCACAAGU-3′ and anti-sense 5′-ACUUGUGGCCGUUUACGUC-3′. The control siRNA bears no homology with any relevant human genes. According to the manufacturer’s instructions, exponentially growing cells were seeded (2.5 × 10^5^ cells per 25 cm^2^ flask) the previous day, in order to reach 30–50% confluency at the time of transfection. Briefly, the transfecting INTERFERin™ reagent (Polyplus Transfection, Strasbourg, France) was added to siRNA (20 nM), diluted in an Opti-MEM® reduced serum medium (Invitrogen) and complex formation was allowed to proceed for 15 min at room temperature before being applied to cells. The following day, the cell media was changed to remove the transfection reagent. At the indicated time, cells were trypsinised and washed with cold PBS. Cell pellets were used directly or stored at −80°C for later use. At least two independent experiments were carried out and typical results are illustrated.

The sodium citrate tribasic pH 7.5 solution was obtained from Sigma Aldrich. 24 H after transfection, cells were treated with citrate.

ABT-737 was provided by Selleckchem (Euromedex, France) and stored as a stock solution in DMSO at −20°C. Exponentially growing cells were exposed to citrate in complete growth medium for 24 H then incubated for 24 H with or without an ABT-737 supplement.

### Nuclear morphology study

After treatment, detached cells were collected separately and adherent cells were dissociated by trypsin/EDTA. The cells were then pooled and collected on a polylysine-coated glass slide by cytocentrifugation, fixed in an ethanol/chloroform/acetic acid solution (6:3:1) and incubated for 15 min at room temperature with a 1 μg/mL DAPI aqueous solution (Boehringer Mannheim, Germany). Slides were thereafter extensively washed in distilled water, mounted in Mowiol (Calbiochem, Meudon, France) and analysed under a fluorescence microscope.

### Analysis of cellular DNA content by flow cytometry

Cells were prepared for flow cytometry as detailed previously [14]. Briefly, adherent and detached cells were pooled, washed in PBS and fixed in 70% ethanol, centrifuged then incubated for 30 min at 37°C in PBS. Pellets were then collected and resuspended for staining with propidium iodide (PI) using the DNA Prep Coulter Reagent Kit (Beckman-Coulter, Villepinte France). Samples were then analysed using an EPICS XL flow cytometer (Beckman Coulter, Villepinte, France) equipped with an argon laser at 15 mW, using 488 nm excitation. EXPO 32 software was used for data acquisition.

### RNA isolation and quantitative reverse transcripase-PCR (qRT-PCR)

Total RNA was isolated from ovarian carcinoma cell lines using Trizol (Invitrogen). RNA quantity and quality were assessed using the NanoDropTM 2000 spectrophotometer (Thermo Scientific, Palaiseau, France). qRT-PCR was performed as previously described [15]. Corresponding custom inventoried (ID: Hs00172036_m1 for Mcl-1 and Hs99999905_m1 for GAPDH) TaqMan® Gene Expression. All PCR amplification reactions were carried out in triplicate and detection was performed on an Applied ABI Prism 7500 Fast PCR system (Applied Biosystems). GAPDH was used as a housekeeping reference gene for normalisation.

### Western immunoblotting

Adherent cells were rinsed with deionised water and lysed with a lysis buffer (pH 8.8 30 mmol.L^-1^ Tris buffer containing 6 mol.L^-1^ urea, 2 mol.L^-1^ thiourea, 2% CHAPS, 1X protease inhibitor Mix). Western blot analysis was carried out as previously described [16]. The membrane was either incubated overnight at 4°C in T-TBS-milk 5% with the following primary antibodies: anti-PARP (1: 1000, Cell Signalling Technology, Beverly, USA), anti-Bcl-x_L_ (1: 1000), anti-Mcl-1 (1: 750, Cell Signalling Technology, Beverly), USA anti-actin (1: 5000, Sigma-Aldrich, Saint Quentin Fallavier, France), anti-cleaved caspase 3 (1: 1000, Cell Signalling Technology, Beverly, USA). After three washes with T-TBS and one with TBS, immunoreactivity was detected by enhanced chemiluminescence (ECL Prime kit, GE Healthcare).

## Results

### Effect of citrate alone

**Figure 1:**
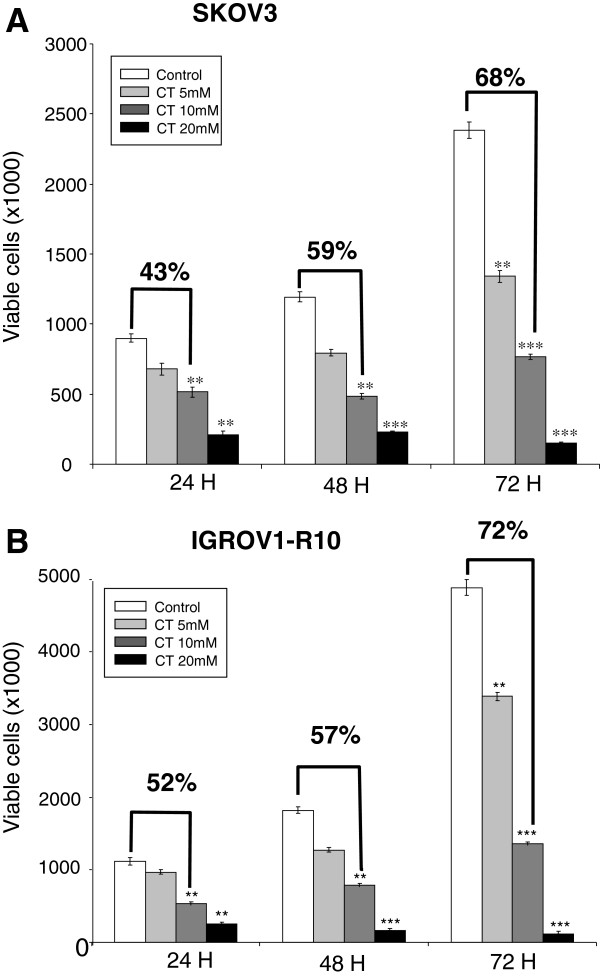
**Effect of citrate on two ovarian cancer cell lines: SKOV3 (A) and IGROV1-R10 (B).** Evolution of viable cells up to 72 H after continuous exposure to 5, 10 and 20 mM of citrate (CT). Exponentially growing SKOV3 cells **(A)** or IGROV1-R10 cells **(B)** were treated with different citrate concentrations in the medium with SVF. The number of viable cells was assessed by the trypan blue exclusion test at various times after treatment. Results are expressed as the mean values of two independent experiments. Analysis of variance was used to determine significance. **: p < 0.05; ***: p < 0.001.

We first studied the kinetic effect of continuous exposure to various concentrations of citrate on two human ovarian carcinoma cell lines, SKOV3 (Figure 1A) and IGROV1-R10 (Figure 1B). As shown in Figure 1A and B, cells exposed to 20 mM citrate demonstrated a high cytotoxic effect from 24 H to 72 H, in contrast with cells exposed to 5 mM citrate. At the 5 mM concentration, the citrate effect is dependent on the time. 72 H after exposure at 5 mM citrate, the inhibition percentage is 68% and 72% in SKOV3 or IGROV1-R10 cells respectively, compared to control cells.

**Figure 2:**
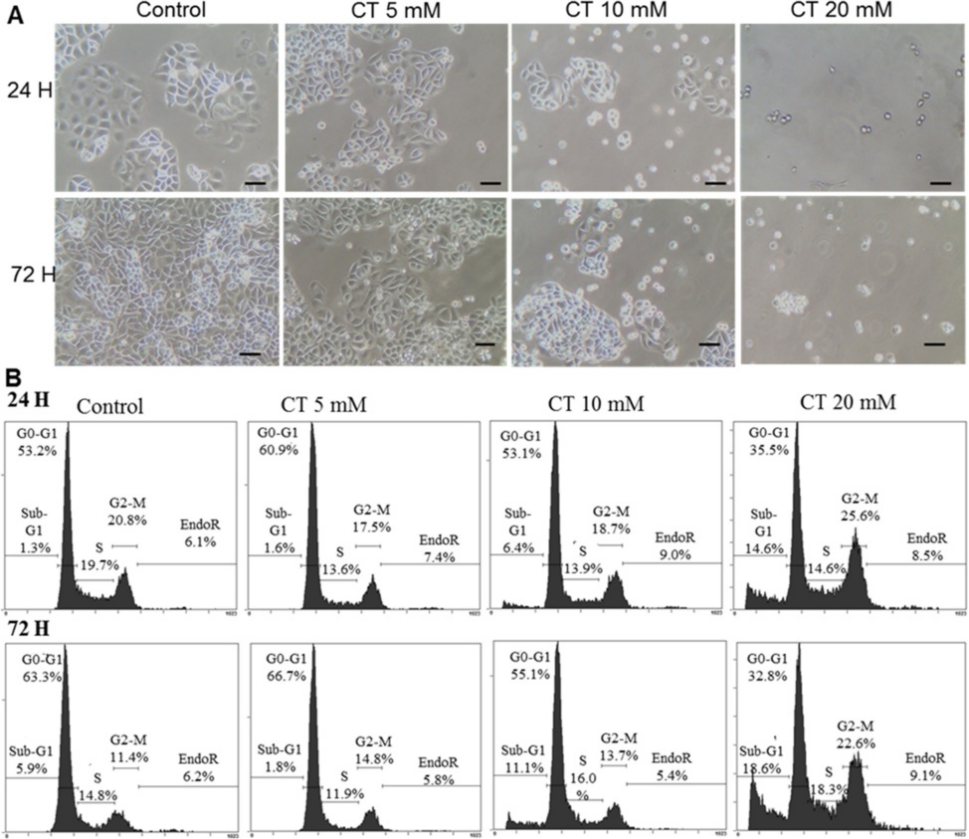
**Effect of various citrate (CT) concentrations (5, 10, 20 mM) in IGROV1-R10 cells after 24 and 72 H exposure. (A)** The morphological features of cell layers were observed by photon microscopy. **(B)** DNA content was determined at 24 H or 72 H by flow cytometry after propidium iodide staining (for each condition, percentages of cells in the different phases of the cell cycle are indicated). Protein expression levels of PARP cleavage and caspase 3 cleavage and Mcl-1 and Bcl-x_L_ on SKVO3 cells **(C)** and on IGROV1-R10 cells **(D)** by western blot analysis in response to citrate at 6 and 24 H. Mcl-1 mRNA expression in SKOV3 cells **(E)** and IGROV1-R10 cells **(F)** treated with citrate for 6 H or 24 H was assessed using real-time quantitative reverse transcription PCR. GAPDH was used as a housekeeping reference gene for normalisation. Each relative mRNA expression level was calculated in comparison to the control cell expression level.

With regard to cellular morphology, distribution in the different phases of the cell cycle and DAPI nuclear staining, SKOV3 cells did not show a marked difference compared to control cells (data not shown). In contrast, IGROV1-R10 cells showed cell rounding, which was concentration-dependent and time-dependent, beginning as early as 24 H (Figure 2A). This phenomenon was associated with a slight accumulation of cells in G2/M phase 24 H after exposure at 20 mM (25.6% 20 mM citrate *vs* 20.8% control) (Figure 2B). Moreover, at this concentration, a subG1 peak appeared, which is characteristic of apoptotic cells, 24 H after treatment by 20 mM citrate; 14.6% were in subG1 compared to 1.3% in control cells. This phenomenon persists over time, 72 H after the same treatment; 18.6% were in subG1 versus 5.9% in control cells (Figure 2B). At the lower concentration of 5 mM citrate, a slight subG1 peak appeared at 24 H (6.4%) and increased at 72 H (11.1%) (Figure 2B).

In these two ovarian carcinoma cell lines, western blot analysis showed that exposure of SKOV3 cells to citrate at 20 mM led to a decrease in the expression of the anti-apoptotic protein Mcl-1, as from 6 H compared to control cells (Figure 2C). This effect was associated with a PARP cleavage, which was only detectable at 24 H (Figure 2C). In the other IGROV1-R10 cell line, we also observed a decrease in Mcl-1 expression after exposure to citrate at 20 mM, with nearly complete extinction of this anti-apoptotic protein at 24 H. At this stage, a slight decrease in Mcl-1 expression is worthy of note (Figure 2D). Cleavage of PARP and caspase 3 were also detected under the same conditions (Figure 2D). In both cell lines, we observed no significant modification in the expression of the other anti-apoptotic protein, Bcl-x_L_, independently of concentrations or time (Figure 2C and D).

The analysis of Mcl-1 mRNA by qRT-PCR showed in SKOV3 cells, 6 H after treatment to 5 – 20 mM citrate, the Mcl 1 mRNA level was not modified. An induction of this transcript was observed at 24 H with the same doses (Figure 2E). In our other cell model, IGROV1-R10, the Mcl-1 mRNA level was slightly induced after 6 H of treatment with 10 to 20 mM citrate. This induction increased at 24 H after exposure to the same doses (Figure 2F). A slight increase in Mcl-1 mRNA at 24 H after exposure to 5 mM citrate in IGROV1-R10 cells was also observed (Figure 2F).

### Effect of concomitant inhibition of Bcl-x_L_ and Mcl-1

#### With siRNA targeting Bcl-x_L_

**Figure 3:**
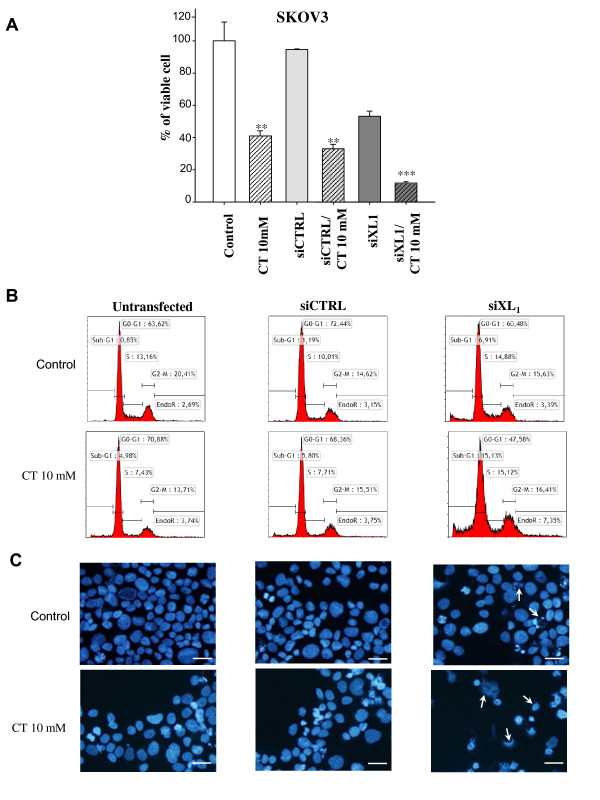
**Effect of siXL1 in response to citrate in the SKOV3 cell line.** Cells were transfected with 20 nM siXL1 24 H before exposure to 10 mM of citrate and cultured for an additional 48 H (i.e. 72 H post transfection). Evolution of viable cells up to 72 H **(A)** in SKOV3. Results are expressed as a percentage of viable cells compared to the 100% control cells. Results are expressed as the mean values of two independent experiments. Analysis of variance was used to determine significance. **: p < 0.05; ***: p < 0.001. **(B)** Cell cycle repartition was studied by flow cytometry after propidium iodide staining at 72 H after transfection in SKOV3 cell lines. **(C)** The nuclear morphology of SKOV3 cells was analysed by DAPI staining at 72 H. Bars 20 μm.

Cells were transfected with siRNA (siXL1 or siCTRL), 24 H before exposure to citrate at 10 mM. As depicted in Figure 3, for SKOV3 cells, citrate at 10 mM and siXL1 significantly reduced (87%) the percentage of viable SKOV3 cells, compared to control cells (Figure 3A). 10 mM Citrate or siXL1 treatment alone inhibits the viability of 59% and 43% of cells respectively. The DNA histogram (Figure 3B) showed a mild increase in the subG1 peak which is a characteristic of apoptotic cells (15% after citrate/siXL1 association vs. 5% after citrate alone or 7% after siXL1 alone). Nuclear staining with DAPI revealed an increased number of remnant cells with “ghost nuclei” (Figure 3C). A combination of siCTRL and citrate did not reveal any difference compared to cells treated with citrate alone (Figure 3A,B,C).

**Figure 4:**
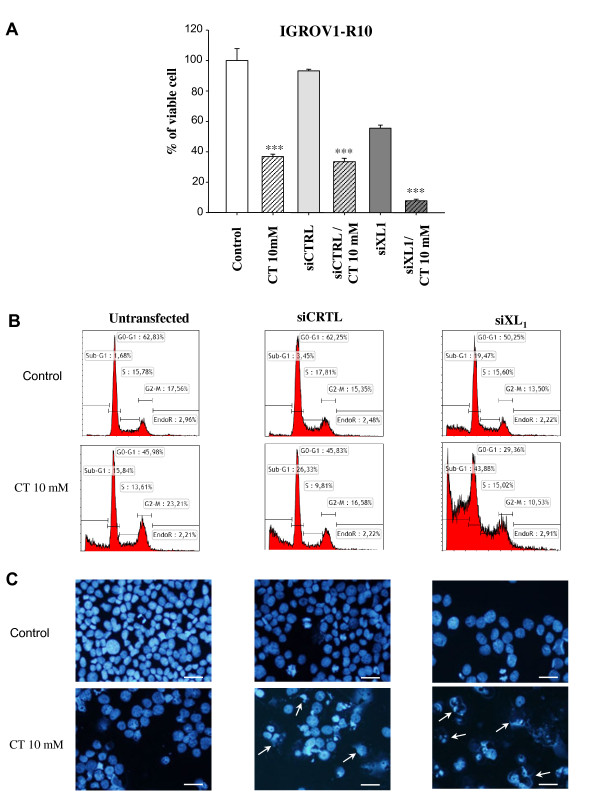
**Effect of siXL1 on response to citrate in the IGROV1-R10 cell line.** Cells were transfected with 20 nM siXL1 24 H before exposure to 10 mM of citrate and cultured for an additional 48 H (i.e. 72 H post transfection). **(A)** Evolution of viable cells up to 72 H in IGROV1-R10. Results are expressed as a percentage of viable cells compared to the 100% control cells. Results are expressed as the mean values of two independent experiments. Analysis of variance was used to determine significance. **: p < 0.05; ***: p < 0.001. **(B)** Cell cycle repartition was studied by flow cytometry after propidium iodide staining at 72 H after transfection in IGROV1-R10 cell lines. **(C)** Nuclear morphology of IGROV1-R10 cells was analysed by DAPI staining at 72 H. Bars 20 μm.

For IGROV1-R10 cells (Figure 4), citrate at 10 mM associated with siXL1 led to a 91% reduction in viable cells compared with the control, which was significantly more important than citrate alone or siXL1 alone, which respectively led to a reduction of 62%, and 43%. 72 H after exposure to citrate and siXL1, IGROV1-R10 cells showed significant cell rounding and cell death in the flask culture (data not shown). This phenomenon was confirmed by the DNA histogram, which revealed a strong subG1 peak (44% of cells) at this stage and under these treatment conditions (siXL1 and citrate) (Figure 4B). DAPI staining showed few normal nuclei, whereas numerous “ghost nuclei” were observed (Figure 4C).

**Figure 5:**
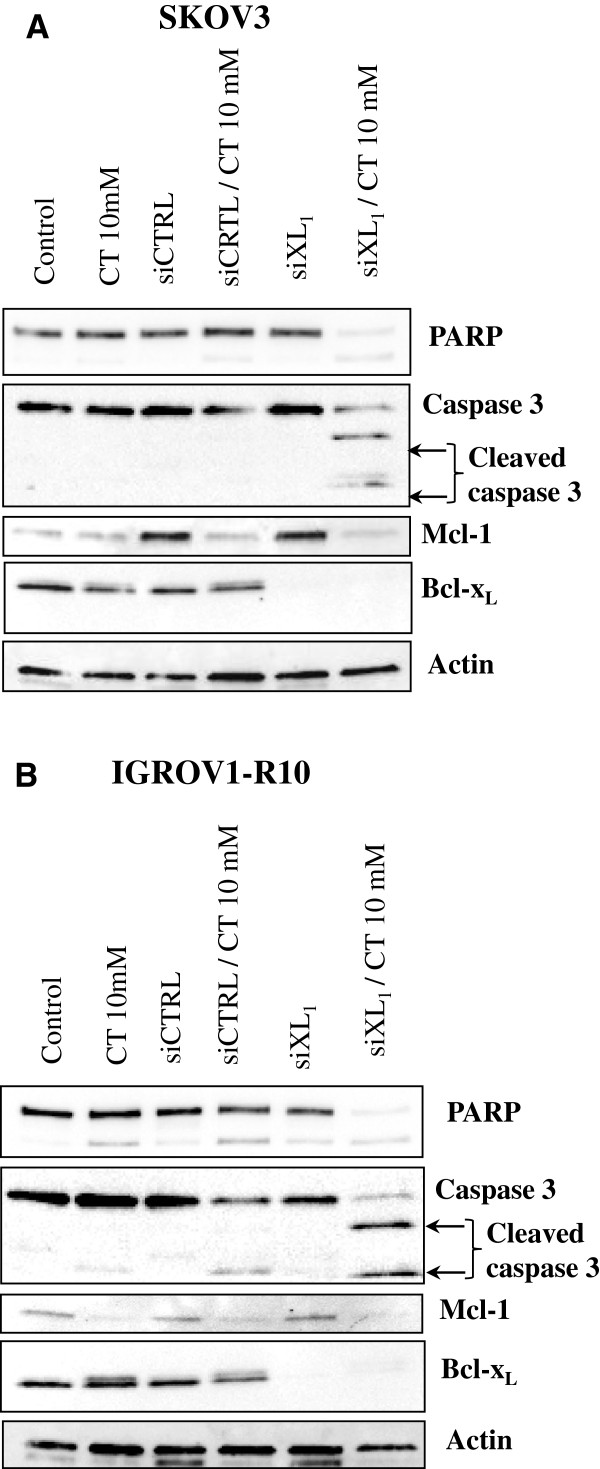
**Protein expression in SKOV3 (A) and IGROV1-R10 (B).** Levels of PARP cleavage and caspase 3 cleavage and Mcl-1 and Bcl-x_L_ were detected by western blot analysis in response to combined treatment with siXL1 and citrate 20 mM, compared with no treatment (control) or siGFP (siRNA control) or citrate alone at 72 H. Actin was used as a loading control.

In both cell lines, western blot analysis confirmed the efficacy of siXL1 to strongly inhibit the expression of Bcl-x_L_ up to 72 H after transfection, compared with SKOV3 cells (Figure 5A) and IGROV1-R10 cells (Figure 5B) transfected with siCTRL. In both cell lines, we also confirmed that 10 mM citrate reduced the expression of Mcl-1 48 H after treatment. As shown in Figure 5A and B, strong activation of caspase 3 leading to the total cleavage of PARP was only observed after cell exposure to combined treatment.

#### ABT-737 treatment

**Figure 6:**
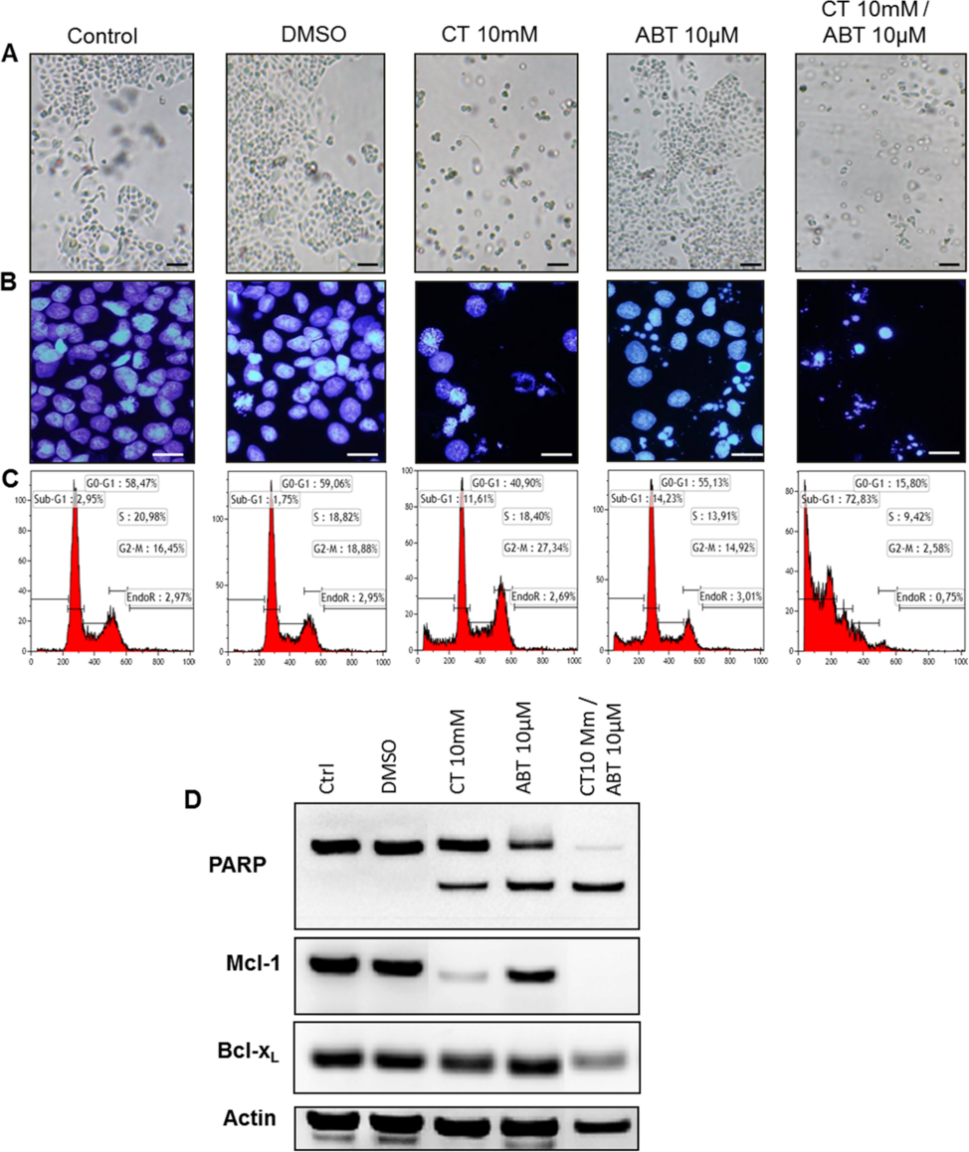
**Effect of citrate on response to ABT-737 in IGROV1-R10 cell lines.** Cells were treated with 10 mM citrate 24 H before exposure to 10 μM of ABT-737 and cultured for an additional 24 H (i.e. 48 H post citrate exposure). **(A)** Morphological features of cell layers at 72 H after citrate exposure. **(B)** Nuclear morphology of IGROV1-R10 cells was analysed by DAPI staining at 72 H. Bars 20 μm. **(C)** Cell cycle repartition was studied by flow cytometry after propidium iodide staining at 72 H after transfection in IGROV1-R10 cell lines. (For each condition, percentages of cells in the different phases of the cell cycle are indicated). **(D)** Protein expression levels of PARP cleavage, Mcl-1 and Bcl-x_L_ on IGROV1-R10 cells. Actin was used as a loading control.

We added ABT-737, a BH3 mimetic compound to block Bcl-x_L_ activity, to the cell culture 24 H after citrate exposure. Since efficacy was more marked in IGROV1-R10 cells, we focused our results on this cell line. This combined treatment induced strong cytotoxicity, with very few viable cells on the flask culture (Figure 6A). Nuclear morphology and the DNA histogram revealed very strong apoptotic cell death, with numerous nuclear fragments and DNA remnants (73% in subG1 peak; Figure 6C). This combined treatment led to the almost complete disappearance of cells in G2/M (2.5%), with a drastic reduction in G1 phase cells (15%) (Figure 6C). DAPI nuclear staining confirmed the DNA histogram; we exclusively observed “ghost nuclei” in response to citrate/siXL, whereas citrate or siXL1 alone induced some apoptotic morphologies and normal nuclei (Figure 6B). In contrast, cells exposed to citrate or ABT −737 alone did not demonstrate the same efficacy in inducing cell death (Figure 6C). Citrate 10 mM led to a stronger reduction in Mcl-1 expression when associated with ABT-737, compared with citrate alone (Figure 6D). This combined treatment led to a cleavage of PARP and a reduction in Bcl-x_L_ expression (Figure 6D).

## Discussion

In the absence of effective chemotherapy, other anti-cancer treatment modalities (surgery, radiotherapy) are most often doomed to failure, as demonstrated for ovarian cancers, for which 5-year survival rates are generally poor, as for many other solid cancers. Hypoxic cells are believed to play an important role in chemoresistance [17-19], since these cells may adopt resistance strategies, such as enhanced glycolysis, glutaminolysis, overexpression of anti-apoptotic factors, etc., to survive harsh chemotherapy conditions [20,21].

Overexpression of anti-apoptotic proteins such as Bcl-x_L_ and Mcl-1 have been reported to greatly contribute to cell survival and drug resistance in various human cancers [22-24]. Concomitant inhibitory strategies targeting these proteins have consequently been shown to overcome drug resistance [11;14].

With this in mind, Bcl-x_L_ inhibitors, such as ABT-737, are currently under clinical evaluation as BH3 mimetic compounds, and few or no specific inhibitors of Mcl-1 are available [45;46]; the discovery of new Mcl-1 inhibitors is therefore of crucial importance. Since we observed that citrate, an inhibitor of PFK, reduces Mcl-1 expression [11], we chose to test citrate in association with a specific siRNA targeting Bcl-x_L_ or ABT-737.

Our results confirm, for the two ovarian cancer cell lines studied, the efficiency of citrate in decreasing Mcl-1 expression, as we have previously described on other cell lines [13,25]. In contrast, citrate demonstrated no significant effect on Bcl-x_L_ expression. The decrease in Mcl-1 protein expression but not Bcl-x_L_ could be due to the activation of caspase 3 as described in other studies [32,33] or by proteasome activity which degrades the Mcl-1 protein after ubiquitylation [27-30]. A recent study reported that the inhibition of glycolysis using 2-deoxy-D-glucose (2DG) induced intracellular ATP depletion which led to specific down regulation of Mcl-1 through the translational control [34].

Concomitant inhibition of Mcl-1 and Bcl-x_L_ is a highly effective strategy to destroy chemoresistant ovarian cancer cells, in accordance with previous studies we have conducted on cancer cells from other cancer sites [11;14;33]. In the two human ovarian cell lines studied, all cells were totally destroyed after 72 H of exposure to treatment associating siXL1 and citrate 10 mM, compared to the administration of one product alone (Figures 3 and 4). We chose to expose cells to citrate 24 h after siXL1 transfection, to allow the siXL1 sufficient time to play its role. In contrast, since the blockage of Bcl-x_L_ activity by ABT-737 is very rapid, occurring within two hours, we chose to administrate citrate before ABT treatment. This association of citrate with ABT-737 seemed more efficient than the combination using siRNA targeting Bcl-x_L,_ and led to almost complete cell death with nuclear apoptotic fragmentation and complete cleavage of PARP. Furthermore, the number of cells in G1 and G2/M phases, likely to resume the cell cycle, was strongly diminished. The mechanisms of cellular death in response to citrate exposure remain to be further investigated. A number of hypotheses can be put forward: - an increase in N-alpha-acetylation of proteins such as caspases, sensitising these cells to apoptosis, given that citrate is the donor of acetyl in cells through ACLY activity [35], an activating process that could be initiated at apical caspase 8 and 2 level [25]; a reduction in ATP synthesis due to glycolysis inhibition by citrate effect, which in turn activates the AMPK pathway, resulting in inhibition of the mTOR pathway [28,30,34]. This pathway could inhibit Mcl-1 expression via two mechanisms: - firstly, Mcl-1 could be phosphorylated by activated GSK3 beta and then degraded by proteasome after ubiquitylation [27-30]; - secondly, blockage of 4E-BP1 resulting in the inhibition of Mcl-1 translation [54;55]. According to our results, the first hypothesis appears the most likely. This molecular aspect remains to be elucidated.

In conclusion, the effect of citrate on Mcl-1 expression could usefully be associated with inhibitors of Bcl-x_L_, since this strategy demonstrates a strong anti-cancer effect on chemoresistant ovarian cancer cells. Given that few or no specific inhibitors of Mcl-1 are currently available, the use of citrate targeting Mcl-1 could be of major interest for it could contribute towards the destruction of chemoresistant cancer cells.

## Abbreviations

CDDP: Cisplatin [cis-diamino-dichloro-platinum (II)]; CT: Citrate; DAPI: 4′,6-diamidino 2-phenylindole; PFK: Phosphofructokinase; PI: Propidium iodide; siRNA: Small interfering RNA; siXL1: SiRNA targeting Bcl-x_L_; TCA: Tricarboxylic acid cycle.

## Competing interests

The authors declare that they have no competing interests.

## Authors’ contribution

HL and PI drafted the manuscript. All authors read and approved the final manuscript.
